# NCoR1 restrains thymic negative selection by repressing *Bim* expression to spare thymocytes undergoing positive selection

**DOI:** 10.1038/s41467-017-00931-8

**Published:** 2017-10-16

**Authors:** Jianrong Wang, Nanhai He, Na Zhang, Dexian Quan, Shuo Zhang, Caroline Zhang, Ruth T. Yu, Annette R. Atkins, Ruihong Zhu, Chunhui Yang, Ying Cui, Christopher Liddle, Michael Downes, Hui Xiao, Ye Zheng, Johan Auwerx, Ronald M. Evans, Qibin Leng

**Affiliations:** 1 CAS Key Laboratory of Molecular Virology & Immunology, Unit of Immune Regulation, Institut Pasteur of Shanghai, Chinese Academy of Sciences; University of Chinese Academy of Sciences, Shanghai, 200031 China; 2 Gene Expression Laboratory, Salk Institute for Biological Studies, La Jolla, CA 92037 USA; 3 Obstetrics and Gynecology Hospital, Shanghai Key Laboratory of Female Reproductive Endocrine-related Disease, the Academy of Integrative Medicine, Fudan University, Shanghai, 200011 China; 4 Storr Liver Centre, Westmead Millennium Institute, Sydney Medical School, University of Sydney, Sydney, NSW 2006 Australia; 5 Immunobiology and Microbial Pathogenesis Laboratory, The Salk Institute for Biological Studies, La Jolla, CA 92037 USA; 6 Laboratory of Integrative and Systems Physiology, École Polytechnique Fédérale de Lausanne, Station 15, Lausanne, CH-1015 Switzerland

## Abstract

Thymocytes must pass both positive and negative selections to become mature T cells. Negative selection purges thymocytes whose T-cell receptors (TCR) exhibit high affinity to self-peptide MHC complexes (self pMHC) to avoid autoimmune diseases, while positive selection ensures the survival and maturation of thymocytes whose TCRs display intermediate affinity to self pMHCs for effective immunity, but whether transcriptional regulation helps conserve positively selected thymocytes from being purged by negative selection remains unclear. Here we show that the specific deletion of nuclear receptor co-repressor 1 (NCoR1) in T cells causes excessive negative selection to reduce mature thymocyte numbers. Mechanistically, NCoR1 protects positively selected thymocytes from negative selection by suppressing *Bim* expression. Our study demonstrates a critical function of NCoR1 in coordinated positive and negative selections in the thymus.

## Abstract (web summary)

Thymocytes are screened by two processes, termed positive and negative selections, which are permissive only for immature thymocytes with intermediate avidity to the selecting ligands. Here the authors show that the nuclear receptor NCoR1 suppresses Bim1 to inhibit negative selection and promote thymocyte survival.

## Introduction

Thymocytes bearing αβ T-cell receptors (TCR) must undergo positive and negative selections before becoming mature T cells^1–5^. Positive selection occurs when thymocytes express TCRs with intermediate affinity to self-peptide MHC complexes (self pMHC) that are presented on cortical thymic antigen-presenting cells. Positive selection helps thymocytes to survive and become mature CD4^+^ or CD8^+^ single-positive (SP) T cells^4, 5^. By contrast, negative selection eliminates CD4^+^CD8^+^ double-positive (DP) or SP thymocytes expressing TCRs with high affinity to self pMHCs^3^. Although the strength of the self pMHC-TCR interaction determines the fate of thymocytes undergoing selection, the mechanisms ensuring the survival of thymocytes that are undergoing positive selection remain unclear.

The transcriptional repressor, nuclear receptor co-repressor 1 (NCoR1), has important functions in metabolism, cellular development, and cancer^6, 7^. NCoR1 binds to nuclear receptors, including thyroid hormone receptor, retinoic acid receptor and glucocorticoid receptor (GR), and further recruits histone deacetylases (HDAC) to regulate the expression of nuclear receptor target genes^6, 8^. Consistent with the function of GR in thymocyte selection^9, 10^, NCoR1 was also shown to have an important function in early T-cell development^11^. Evidently, germline deletion of NCoR1 results in embryonic lethality^11^ and smaller fetal thymi than those of wild-type (WT) controls. Further analysis by fetal thymic organ culture reveals an early blockade of thymocyte development at the CD4^−^CD8^−^ double-negative (DN) 3 (CD25^+^CD44^−^) stage. However, due to the embryonic lethality induced by NCoR1 deficiency, it is still unclear whether this blockade reflects an intrinsic defect attributable to the loss of NCoR1 in thymocytes, or extrinsic effects from the thymic microenvironment. In addition, how NCoR1 regulates thymocyte development at later stages, particularly during positive and negative selection, remains unknown.

To better define the function of NCoR1 in thymocyte development, we generate T-cell-specific NCoR1-knockout mice. We find that NCoR1 deficiency impacts positive selection by inducing apoptosis in activated thymocytes, resulting in reduced mature T cells in the thymus and periphery of NCoR1-deficient mice. In addition, NCoR1 deficiency upregulates the expression of *Bim*, a key mediator of negative selection^12^. *Bim*-knockout rescues the thymocyte developmental defect in NCoR1-deficient mice, suggesting that *Bim* may be involved in this positive selection defect. Mechanistically, NCoR1 represses *Bim* expression by binding to *Bim* promoter and promoting histone deacetylation. Our data reveal a transcriptional mechanism that protects thymocytes with ongoing positive selection from negative selection, and further suggest that NCoR1 is essential for efficient thymocyte development and optimal peripheral T-cell homeostasis.

## Results

### NCoR1 is essential for the late-stage thymocyte development

To better understand how NCoR1 regulates thymocyte development, we first analyzed *Ncor1* mRNA expression in different subsets of thymocytes (as well as other tissues). The expression levels of *Ncor1* in the thymus were comparable to those in the liver and muscle (Supplementary Fig. 1a). Among the thymocyte subsets, CD4^+^CD8^+^ DP cells expressed a relatively higher level of *Ncor1* than CD4^−^CD8^−^ DN, CD4^+^ or CD8^+^ SP thymocytes (Supplementary Fig. 1b), indicating that NCoR1 may also play an important role in late-stage thymocyte development, similar to its function in early thymocyte development^11^.

**Fig. 1:**
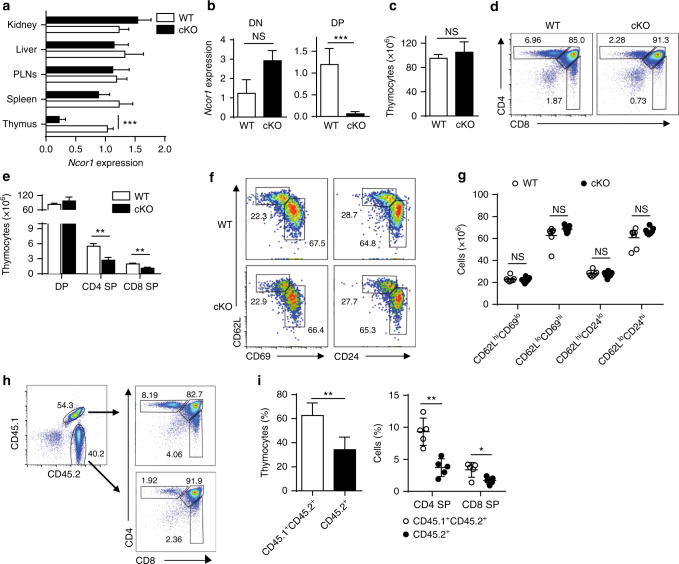
Late-stage thymocyte development is defective in NCoR1-deficient mice. **a**, **b** Quantitative RT-PCR analysis of *Ncor1* mRNA levels in indicated tissues (**a**, *n* = 5), and sorted CD4^−^CD8^−^ double-negative (DN) thymocytes (*n* = 3) and CD4^+^CD8^+^ double-positive (DP) (*n* = 6) (**b**) thymocytes from NCoR1-deficient (cKO) and wild-type (WT) mice. **c**, **d** Total cell numbers (**c**) and surface expression of CD4 and CD8 (**d**) on thymocytes from cKO (*n* = 5) and WT (*n* = 5) mice detected by flow cytometry. **e** Absolute cell numbers of DP, CD4^+^ or CD8^+^ single-positive (SP) thymocytes from cKO and WT mice (*n* = 5 for each group), as the product of total thymocytes multiplied by the percentage of cells found in that population. **f** Expression profiles of CD62L vs. CD69 or CD24 on cKO (*n* = 3) and WT (*n* = 3) CD4 SP thymocytes detected by flow cytometry. **g** Cell numbers of each subpopulation gated in (**f**). **h** Expression of CD4 and CD8 (right) from mix-bone marrow chimeras generated from CD45.1^+/−^ WT and CD45.2^+^ cKO (left) donors (*n* = 4 to 5). **i** Quantification of each subpopulation gated in (**h**). The data are representative of two independent experiments (**a**, **b**, **h**) or three independent experiments (**c**–**f**). Statistical significance was analyzed using the two-tailed Student’s *t* test (*P < 0.05; **P < 0.01; ***P < 0.001; NS, not significant)

To investigate the cell-type-specific function of NCoR1 in thymocyte development, we generated thymocyte-specific knockout mice by crossing *Ncor1* ^*flox/flox*^ mice^13, 14^ with *Lck-Cre-recombinase* (*Cre)* or *CD4-Cre* transgenic mice (to generate cKO or 4KO mice, respectively). *Ncor1* ^*flox/flox*^ mice that do not express *Cre* served as WT controls. Both *Ncor1* mRNA and protein levels were decreased by ~70% in whole thymi of NCoR1-deficient mice compared with WT mice (Figs. 1a and 5d). Regarding the subsets of thymocytes, *Ncor1* expression in the DN thymocytes of cKO mice was similar to that in WT mice, but its expression was decreased (by ~95%) in cKO DP thymocytes (Fig. 1b). NCoR1 deficiency did not significantly alter the total thymocyte number in the thymi of cKO mice (Fig. 1c). By contrast, both CD4 SP and CD8 SP cells from cKO mice were decreased by ~50% not only proportionally but also in total cell numbers compared with WT or *Lck-Cre* only mice (Fig. 1d, e and Supplementary Fig. 2a, b). A similar reduction in CD4 SP and CD8 SP thymocytes was also observed in 4KO mice (Supplementary Fig. 3). Further analysis of the maturation status of CD4 SP thymocytes revealed similar proportions of mature (CD62L^hi^CD69^lo^ or CD62L^hi^CD24^lo^) and immature (CD62L^lo^CD69^hi^ or CD62L^lo^CD24^hi^) subsets in cKO mice and WT mice (Fig. 1f, g), indicative of relatively normal SP thymocyte maturation. Thus, NCoR1 deficiency leads to a defect in the transition from DP to SP thymocytes.

To identify whether the defect in NCoR1-deficient mice is intrinsic or extrinsic, we generated chimeric mice by reconstituting irradiated *Rag1* ^−/−^ mice with mixed cKO (CD45.2^+^) and WT (CD45.1^+^CD45.2^+^ or CD45.1^+^) bone marrow (BM) cells at a ratio of 1:1. Analysis of the thymocytes at 6 weeks post-reconstitution revealed a significantly lower proportion of cKO compared with WT (Fig. 1h, i). The percentages of both CD4 SP and CD8 SP thymocytes of cKO mice were more drastically decreased compared with those of WT mice (Fig. 1h, i). Thus, NCoR1 is intrinsically required for the developmental transition from DP to SP thymocytes. Collectively, these data strongly suggest that NCoR1 plays a critical role in the late stages of T-cell development.

### NCoR1 deficiency leads to a defect in positive selection

**Fig. 2:**
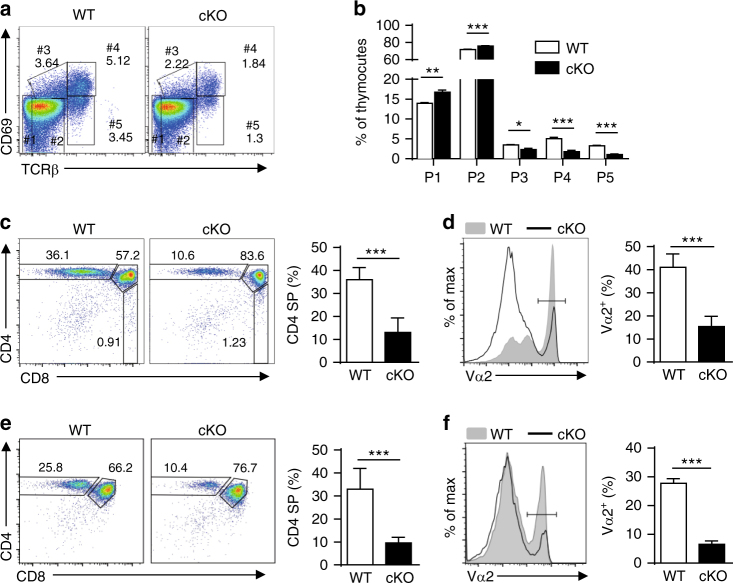
NCoR1 deficiency leads to a blockade in positive selection. **a** The surface expression of CD69 and TCRβ detected by flow cytometry on NCoR1-deficient (cKO) and wild-type (WT) thymocytes (*n* = 5 for each group). **b** Quantification of each subpopulation gated in (**a**). **c**, **e** The expression profiles of CD4 and CD8 (left) on total thymocytes from cKO and WT OT-II TCR-transgenic mice in the *Rag1* ^+/+^ (**c**, *n* = 4) or *Rag1* ^−/−^ (**e**, *n* = 3) background. The right panel shows the percentages of CD4^+^ single-positive (SP) thymocytes gated in the left panel. **d**, **f** The expression of TCR Vα2 detected by flow cytometry on total thymocytes from cKO and WT OT-II TCR-transgenic mice in *Rag1* ^*+/+*^ (**d**, *n* = 4) or *Rag1* ^−*/*−^ (**f**, *n* = 3) background. The right panel shows the quantification of TCR Vα2^+^ thymocytes gated in the left panel. The data are representative of three independent experiments (**a**–**f**). Statistical significance was analyzed using the two-tailed Student’s *t* test (*P < 0.05; **P < 0.01; ***P < 0.001)

To further explore the stage of thymocyte development that was impaired in NCoR1-deficient mice, we stratified the thymocytes into five developmental stages based on the expression of TCRβ and CD69 as previously reported^15, 16^ (Fig. 2a, b). NCoR1-deficient mice exhibited slightly but significantly higher numbers of the two early-stage populations, TCRβ^lo^CD69^lo^ cells (population 1) and TCRβ^int^CD69^lo^ cells (population 2), compared with WT mice. Since TCRβ^int^CD69^lo^ cells are primarily pre-selection DP cells, NCoR1 deficiency does not seem to affect the development of pre-selection thymocytes. However, NCoR1-deficient mice had significantly fewer CD69^+^ thymocytes, in which thymic selection is supposed to occur, including both TCRβ^int^ (population 3) and TCRβ^hi^ (population 4) populations. Consequently, in the absence of NCoR1, the more mature TCRβ^hi^CD69^lo^ cells (population 5, primarily SP cells), were reduced by approximately 60% (Fig. 2a, b). It has also been reported that when thymocytes contact self pMHCs on cortical epithelial cells, CD5 expression is upregulated, reflecting the affinity of TCR-self pMHC self pMHC-TCR complexes and the strength of the TCR signaling^17, 18^. We found that, similar to the overall decreased expression of CD69 (Fig. 2a), CD5 expression also significantly declined in NCoR1-deficient thymocytes (Supplementary Fig. 4a). Further analysis of DP thymocytes revealed that NCoR1 deficiency mainly affected thymocytes undergoing positive selection, because the reduction of CD5 expression only occurred in CD69-positive subpopulations but not in CD69-negative subpopulations (Supplementary Fig. 4b). Taken together, these data suggest that NCoR1 deficiency disrupts the selection process of thymocytes upon contact with self- pMHCs.

To evaluate whether NCoR1 deficiency affects positive selection, we bred MHC class II-restricted OT-II TCR-transgenic mice with cKO mice. CD4 SP thymocytes of NCoR1-deficient mice were decreased by ~60% compared with those of WT mice (Fig. 2c). TCR Vα2^+^ thymocytes in NCoR1-deficient mice decreased ~60% compared with those in WT transgenic mice (Fig. 2d). In addition, CD69 expression was decreased by ~70% in NCoR1-deficient OT-II mice (Supplementary Fig. 4c). A similar reduction was observed in OT-II mice carrying a RAG1 deficiency, in which no other rearranged TCRs are expressed (Fig. 2e, f). Taken together, these findings suggest that NCoR1 is required for positive selection.

The selection of thymic Foxp3 T regulatory cells (tTreg) and invariant natural killer T (iNKT) cells differs from that of conventional CD4 SP thymocytes because tTregs recognize self- pMHC with a higher affinity than conventional CD4 SP thymocytes and iNKT cells recognize lipid antigens that are presented by CD1d. Therefore, we also analyzed the effects of the NCoR1 deficiency on tTregs and iNKT cells. As shown in Supplementary Fig. 5, both tTregs and iNKT cells in cKO mice were decreased by ~50% compared with WT mice. The decrease in tTregs mainly resulted from the overall reduction of CD4 SP thymocytes, because the proportion of tTregs in CD4 SP thymocytes of cKO mice was comparable to that of WT mice (Supplementary Fig. 5b). This result indicates that NCoR1 deficiency causes a general defect in thymocyte selection.

### NCoR1 deficiency increases thymocyte apoptosis

**Fig. 3:**
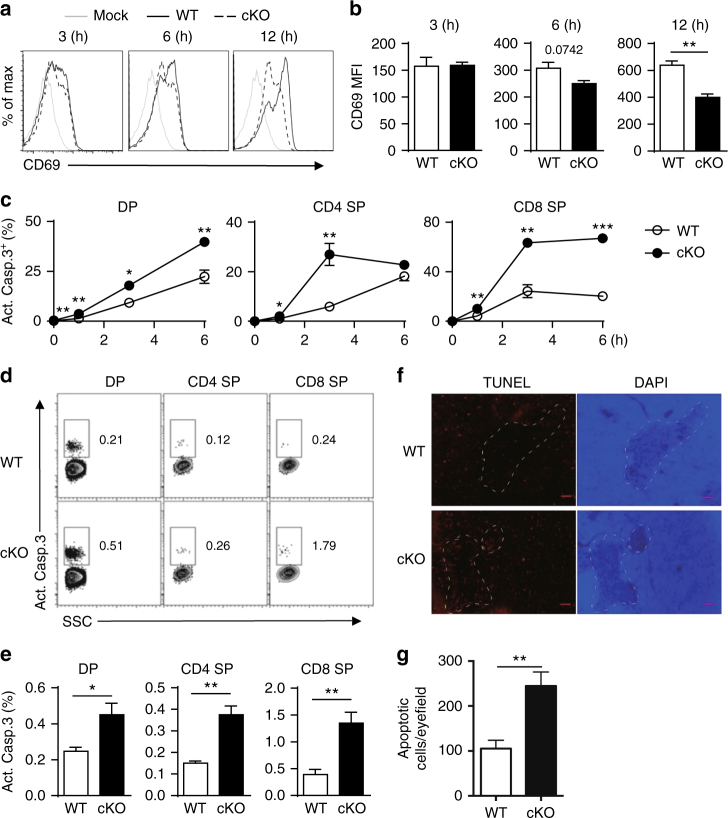
Deletion of NCoR1 causes elevated thymocyte apoptosis. **a** Flow cytometry analysis of CD69 expression on sorted in vitro-stimulated CD4^+^CD8^+^ double-positive (DP) thymocytes from NCoR1-deficient (cKO) and wild-type (WT) mice (*n* = 3). **b** The mean fluorescence intensity of CD69 expression in (**a**). **c** Intracellular levels of the active caspase-3 (Act.Casp.3) detected by flow cytometry in cKO and WT thymocytes after in vitro TCR stimulation for indicated amounts of time (*n* = 3 for each group). **d**, **e** Intracellular levels of the active caspase-3 (Act.Casp.3) detected by flow cytometry in freshly isolated thymocytes (*n* = 4 to 5). **f** TUNEL staining of the thymus sections of cKO and WT mice (*n* = 10). The scale bars indicate 100 μm. **g** Quantification of the apoptotic cells in (**f**). The data are representative of two independent experiments (**a**–**e**). Statistical significance was analyzed using the two-tailed Student’s *t* test (*P < 0.05; **P < 0.01; ***P < 0.001)

Attenuated TCR signaling, particularly reduced ERK pathway signaling, leads to impaired positive selection^15, 16, 19–21^. To determine whether defective TCR signaling is involved in the decreased positive selection in NCoR1-deficient mice, we stimulated thymocytes with anti-TCRβ and anti-CD28 antibodies, and examined the activation of TCR signaling pathways. We observed similar phosphorylation of PLCγ1, Erk1/2 and p38MAPK in NCoR1-deficient and WT thymocytes upon TCR stimulation (Supplementary Fig. 6), suggesting that TCR signaling was not defective in the NCoR1-deficient thymocytes. In addition, the expression level of CD69, an early T-cell activation marker, was comparable between WT and NCoR1-deficient thymocytes in the early stage of in vitro TCR stimulation (Fig. 3a, b), further strengthening the idea that defective positive selection in NCoR1-deficient mice does not result from a defect in TCR signaling.

However, in the late stage of in vitro TCR stimulation, we noticed that CD69 expression on NCoR1-deficient thymocytes was significantly lower than that on WT thymocytes (Fig. 3a, b). As CD69 expression was not affected by the NCoR1 deficiency in the early stage of TCR activation, we therefore sought to examine whether the reduced CD69 expression in the late stage of TCR activation resulted from a loss of activated thymocytes. We found an ~3-fold increase in apoptotic cell numbers in NCoR1-deficient DP and SP thymocytes compared with WT mice by measuring activated caspase-3 (Fig. 3c and Supplementary Fig. 7a). Noticeably, apoptotic thymocytes tended to have significantly lower CD69 expression regardless of the NCoR1 deficiency (Supplementary Fig. 7b). Annexin V staining also revealed an increase in apoptosis in NCoR1-deficient thymocytes (Supplementary Fig. 7c, d). Further analyses of ex vivo thymocytes and tissue sections also revealed a significant increase in apoptosis in NCoR1-deficient thymocytes (Fig. 3d–g). These findings suggest that dysregulated apoptosis contributes to the decline in positive selection in NCoR1-deficient mice.

### NCoR1 suppresses negative selection

**Fig. 4:**
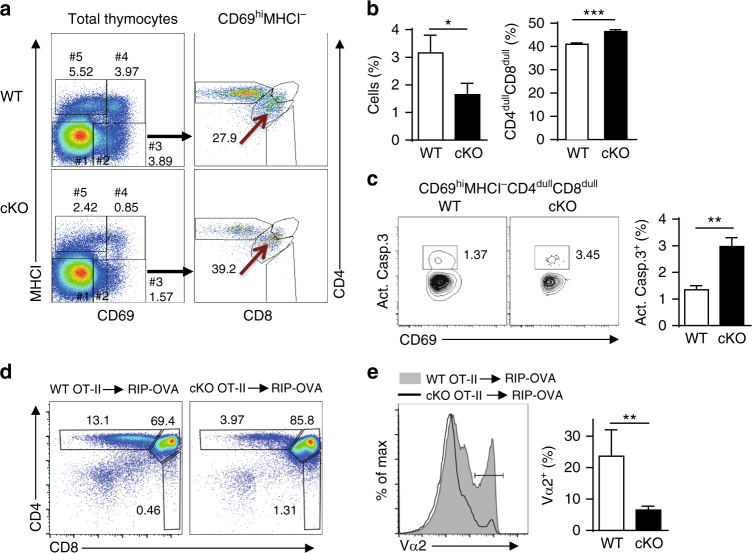
NCoR1 is required to prevent excessive negative selection. **a** Flow cytometry analysis of 5 subpopulations (left) by MHCI and CD69 expression on total NCoR1-deficient (cKO) and wild-type (WT) thymocytes (*n* = 3 to 5). The right panel shows CD4 and CD8 expression on gated population #3. **b** The proportion of population #3 (left) and CD4^dull^CD8^dull^ subsets in population #3 (right). **c** Intracellular levels of Act.Casp.3 detected by flow cytometry in the CD69^hi^MHCI^−^CD4^dull^CD8^dull^ subsets from cKO and WT mice (*n* = 4 to 5 for each group). **d** Flow cytometry analysis of CD4 and CD8 expression on total thymocytes 6 weeks after transferring bone marrow cells from OT-II TCR-transgenic cKO or WT mice into irradiated rat insulin promoter-ovalbumin (RIP-OVA) transgenic mice (*n* = 4 to 5). **e** TCR Vα2 expression of total thymocytes from the mice in (**d**). The data are representative of three independent experiments (**a**, **b**) or two independent experiments (**c**, **d**). Statistical significance was analyzed using the two-tailed Student’s *t* test (*P < 0.05; **P < 0.01; ***P < 0.001)

Increased apoptosis indicated that excessive negative selection might contribute to the decline in positive selection in NCoR1-deficient mice. As previously reported, the CD69^hi^MHCI^−^CD4^dull^CD8^dull^ subset is a distinct fraction of thymocytes that undergoes negative selection^12^. Thus, we examined the effects of NCoR1 deficiency on this particular cell population. Despite the presence of fewer CD69^+^ thymocytes, NCoR1-deficient mice maintained higher proportions of CD69^hi^MHCI^−^CD4^dull^CD8^dull^ cells than WT mice (Fig. 4a, b and Supplementary Fig. 2c, d), suggesting that more cells undergo negative selection in NCoR1-deficient mice. In addition, the percentage of activated caspase-3-positive cells was ~2-fold higher in this subset in NCoR1-deficient mice than in WT mice (Fig. 4c). Thus, NCoR1 is required to suppress negative selection.

To further corroborate the role of NCoR1 in negative selection, we generated chimeric mice by transferring BM cells of OT-II TCR-transgenic WT and cKO mice into irradiated transgenic mice expressing ovalbumin (OVA) under the control of the rat insulin promoter (RIP). Because OVA is expressed in the thymus in this condition, OT-II thymocytes with OVA-specific TCR are forced to undergo negative selection^5^. Our results showed that the thymi of reconstituted mice with NCoR1-deficient OT-II BM cells had an ~4-fold reduction in the number of CD4 SP and TCR Vα2^+^ thymocytes respectively compared with WT (Fig. 4d, e). These observations indicate that NCoR1 suppresses excessive negative selection.

### NCoR1 binds to the *Bim* promoter and represses *Bim* expression

**Fig. 5:**
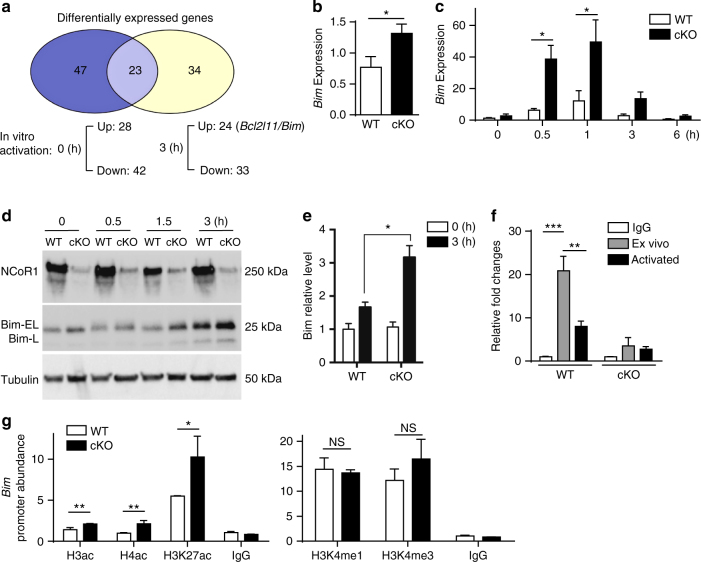
NCoR1 binds to the *Bim* promoter and represses *Bim* expression. **a** RNA-Seq analysis of differentially expressed genes in NCoR1-deficient (cKO) and wild-type (WT) thymocytes with or without activation in vitro. **b**, **c** Quantitative RT-PCR analysis of *Bim* mRNA expression in freshly sorted cKO (*n* = 6) and WT (*n* = 6) CD4^+^CD8^+^ double-positive (DP) thymocytes (**b**), and in total cKO (*n* = 4) and WT (*n* = 4) thymocytes (**c**) after in vitro TCR stimulation for the indicated amounts of time. **d** Immunoblot analysis of NCoR1 and Bim expression levels in extracts of cKO and WT thymocytes with or without stimulation for the indicated amounts of time. **e** Quantification of NCoR1 and Bim expression levels in (**d**). **f** ChIP analysis of the *Bim* promoter by anti-NCoR1 in cKO and WT thymocytes with or without activation in vitro. IgG served as a negative control. **g** ChIP analysis of the *Bim* promoter in cKO and WT thymocytes using the indicted antibodies. The data are representative of two independent experiments (**b**, **c**, **f**, **g**). Statistical significance was analyzed using the two-tailed Student’s *t* test (*P < 0.05; **P < 0.01; ***P < 0.001; NS, not significant)

Given that increased apoptosis is associated with NCoR1 deficiency and that NCoR1 is a transcriptional repressor, we explored whether any proapoptotic genes were de-repressed and hence contributed to the elevated apoptosis of NCoR1-deficient thymocytes. Prior to activation, RNA-Seq analyses revealed 70 genes with significantly changed expression levels between the WT and cKO (28 upregulated and 42 downregulated). Upon activation via TCR ligation, 57 genes were found to be differentially expressed, with 24 upregulated and 33 downregulated genes (Fig. 5a, Supplementary Table 1). Neither CD5 nor CD69 was among the list of differentially expressed genes, likely because the thymocytes expressing these two genes represented only a small fraction of the total thymocytes (Fig. 2a and Supplementary Fig. 4a). Further analysis established that only one proapoptotic gene, *Bim*, was significantly increased, especially in response to in vitro TCR activation. A similar increase in *Bim* expression was found in ex vivo-sorted DP thymocytes (Fig. 5b). Consistent with the expression profiling, the expression level of *Bim* in NCoR1-deficient thymocytes was upregulated 5-fold 30 min after stimulation (Fig. 5c), and western blotting revealed similar increases in Bim protein (Fig. 5d, e and Supplementary Fig. 8a). Bim expression levels were correlated with the TCR signal strength in both WT and cKO mice (Supplementary Fig. 8a). To further corroborate the results, we also examined *Bim* expression levels in nonactivated (CD69 negative) pre-selection population 1 + 2 and activated population 4 + 5 defined in Fig. 2a. The quantitative RT-PCR analysis revealed that the NCoR1 deficiency only affected *Bim* expression in the activated population 4 + 5 but not in the nonactivated pre-selection population (Supplementary Fig. 8b). Although multiple genes have been reported to promote apoptosis of both thymocytes and T cells, and to participate in negative selection^22–25^, we observed no increases in the expression of *Fasl*, *Trail*, *Tnf* and *Bak* in NCoR1-deficient DP thymocytes before or after in vitro activation (Supplementary Fig. 9). Thus, NCoR1 deficiency specifically upregulates *Bim* expression in thymocytes, and this elevated expression of *Bim*, with its known function in negative selection^24–26^, contributes to the increased apoptosis of activated NCoR1-deficient thymocytes.

Next, we sought to examine whether NCoR1 directly controls the transcriptional expression of *Bim*. To achieve this goal, we first performed quantitative RT-PCR using chromatin immunoprecipitated with an antibody against NCoR1 in thymocytes (ChIP-PCR). This assay showed that NCoR1 specifically bound to the *Bim* promoter in WT thymocytes, but not NCoR1-deficient thymocytes (Fig. 5f). In addition, activation of WT thymocytes via TCR ligation led to NCoR1 clearance from the *Bim* promoter (Fig. 5f), consistent with the increases in *Bim* mRNA and protein levels in response to TCR activation (Fig. 5c–e). Taken together, our results indicate that NCoR1 binds directly to the *Bim* promoter to transcriptionally repress its expression.

It is well known that NCoR1 interacts with HDACs such as HDAC3, to promote histone deacetylation. We next analyzed the effects of NCoR1 deficiency on the status of histone acetylation at the *Bim* promoter by the ChIP assay. NCoR1-deficient thymocytes showed increased acetylation of both histones 3 and 4 compared with WT thymocytes. A similar increase in acetylation specifically on histone H3 Lys27 (H3K27ac), a marker of active chromatin, was also observed (Fig. 5g, left panel). By contrast, NCoR1 deficiency did not affect the levels of the inactive chromatin markers, mono- and trimethylated K4-H3 histone (Fig. 5g, right panel). These results suggest that NCoR1 is required to maintain the inactive form of chromatin at the *Bim* promoter by promoting histone deacetylation.

### Elevated *Bim* expression exaggerates negative selection

**Fig. 6:**
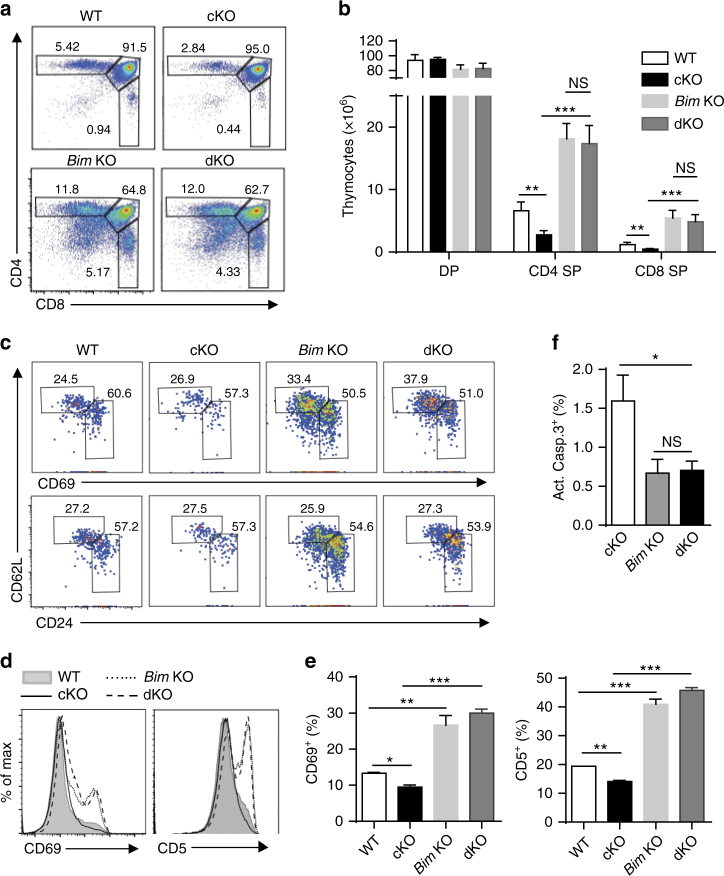
*Bim* expression is responsible for the defective thymocyte development in NCoR1-deficient mice. **a** Flow cytometry analysis of CD4 and CD8 expression on thymocytes from wild-type (WT), NCoR1-deficient (cKO), *Bim* KO, *Ncor1* and *Bim* double-knockout (dKO) mice. **b** The number of cells in each subpopulation gated in (**a**). **c** Expression of CD62L vs. CD69 or CD24 on CD4^+^ single-positive (SP) thymocytes from WT, cKO, *Bim* KO, and dKO mice. **d** The percentages of CD69- and CD5-positive subpopulations in (**c**). **e** Flow cytometry analysis of CD69 and CD5 expression on total thymocytes from WT, cKO, *Bim* KO, and dKO mice. **f** Intracellular levels of the active caspase-3 (Act.Casp.3) in thymocytes activated in vitro for 1 h. The data are representative of two independent experiments (*n* = 2 or 3). Statistical significance was analyzed using the two-tailed Student’s *t* test (*P < 0.05; **P < 0.01; ***P < 0.001; NS, not significant)

To assess the presence of a cause-and-effect relationship between NCoR1 deficiency-induced *Bim* expression and increased apoptosis during T-cell development, we crossed whole-body *Bim* KO mice with the NCoR1-deficient mice to generate mice deficient for both the *Bim* and *Ncor1* genes (dKO). The proportions of CD4 SP and CD8 SP thymocytes in dKO mice increased 4–10 times compared with mice that were NCoR1-deficient only (Fig. 6a, b). Interestingly, the proportions of SP thymocytes and mature SP thymocytes were comparable between *Bim* KO and dKO mice (Fig. 6a, c and Supplementary Fig. 10), suggesting that the effects of the NCoR1 deficiency on thymocytes were mainly dependent on *Bim*. Further analysis of CD69 and CD5 expression in total thymocytes also revealed that the *Bim* knockout was able to rescue both the decreased CD69 and CD5 expression levels in NCoR1-deficient thymocytes (Fig. 6c, d). Noticeably, compared with WT mice, CD5 expression, CD69 expression, and SP proportions were significantly increased in *Bim* KO mice, regardless of NCoR1 deficiency (Fig. 6a, c, d). Taken together, these data strongly suggest that *Bim* deficiency protects against the depletion of both types of thymocytes with ongoing either positive or negative selection.

We also examined the effect of *Bim* deficiency on the apoptosis of activated NCoR1-deficient thymocytes by performing active caspase-3 staining. *Bim* deficiency led to significantly reduced apoptosis in activated thymocytes regardless of NCoR1 deficiency. In addition, the proportions of apoptotic cells in dKO thymocytes did not significantly differ from thymocytes that were deficient for *Bim* alone (Fig. 6f). Overall, our data suggest that the elevated apoptosis of NCoR1-deficient thymocytes is most likely attributable to dysregulated *Bim* expression.

## Discussion

The fate of immature thymocytes is determined based on the strength of the self pMHC-TCR complexes interaction. Only thymocytes bearing TCRs with intermediate affinity to self-pMHCs undergo positive selection and become mature T cells. By contrast, thymocytes expressing TCRs with high affinity to self-pMHCs will undergo negative selection via apoptosis. The transcriptional regulation that protects thymocytes with ongoing positive selection from negative selection remains unclear. In the present study, we showed that T-cell-specific NCoR1-deficient mice experienced a defect in positive selection, leading to a significant reduction of both mature CD4^+^ and CD8^+^ SP T cells. This defect was attributable to increased negative selection. Upregulation of *Bim* expression was associated with increased apoptosis of activated thymocytes. Furthermore, NCoR1 was required for the repression of *Bim* expression in thymocytes by binding to the *Bim* promoter. In addition, strong TCR signaling promoted NCoR1 release from the *Bim* promoter to elicit *Bim* expression. *Bim* gene knockout rescued the defective positive selection in NCoR1-deficient mice. In summary, NCoR1 represses *Bim* expression to protect thymocytes undergoing positive selection from excessive negative selection.

Bim, a proapoptotic molecule, is a key mediator of negative selection during thymocyte development^24–26^. Typically, *Bim* expression is induced only by a negative selection but not by a positive-selection signal^27^. Although weak positive-selection signals also trigger Ca2^+^ flux^27, 28^, only strong TCR activation during negative selection further triggers protein kinase C activation and the subsequent transcriptional induction of *Bim* expression^27^. However, the factors that further mediate protein kinase C-dependent *Bim* transcription in thymocytes were unknown^27^. In this study, we showed that strong TCR signaling released NCoR1 from the *Bim* promoter, leading to the activation of *Bim* transcription in WT thymocytes. Furthermore, NCoR1-deficient OT-II mice on RIP-OVA background exhibited a significant increase in negative selection, indicating that NCoR1 indeed inhibited negative selection. Therefore, NCoR1 is likely a protein kinase C-mediated factor that controls *Bim* expression.

NCoR1 is a large protein with a molecular mass of ~270 kDa and contains a total of three repression domains, two SANT-like domains and three nuclear receptor interaction domains^6, 7, 29^. These different domains enable NCoR1 to serve as a platform for interactions with various chromatin-modifying enzymes and transcription factors or nuclear receptors, as well as the formation of dynamic corepressor complexes^6, 7, 29^. The dynamics and co-occurrence of interacting factors likely contribute to the versatile cell-specific functions of NCoR1 complexes^6, 29^. Studies using cell-specific *Ncor1* knockout mice show that NCoR1 has distinct functions in adipose tissue, muscle, and macrophages through distinct mechanisms^13, 14, 30^, suggesting that NCoR1 has cell- or tissue-specific functions. In the present study, we showed that NCoR1 directly repressed *Bim* expression and inhibited excessive apoptosis of activated thymocytes, optimizing positive selection in the thymus. In addition, NCoR1-deficient (cKO) mice had only ~50% of the numbers of peripheral CD4 and CD8 T cells as WT mice (Supplementary Fig. 11a, b), indicating that NCoR1 may be required for T-cell homeostasis in the periphery. Furthermore, although the proportion of thymic Tregs among CD4 SP thymocytes was comparable to that of WT mice, the proportion of peripheral Tregs among CD4 T cells was increased by ~50% (Supplementary Fig. 11c, d). This result suggests that NCoR1 may also be involved in the homeostasis of peripheral Tregs. Altogether, NCoR1 most likely has diverse functions in different immune cells.

NCoR1 recruits different HDAC and forms complexes in a context-dependent manner^6, 7, 11^. Several studies have revealed that HDAC3 is a major enzyme contributing to the repressive activity of NCoR1^6, 31–33^. To our surprise, thymocyte development is not defective in HDAC3-floxed *CD4-Cre-recombinase* (*Cre*) mice^34^, which is different from the NCoR1-floxed *CD4-Cre* mice in the present study. Nevertheless, HDAC3-floxed *Lck-Cre* mice are phenotypically similar to NCoR1-floxed *Lck-Cre* mice, exhibiting significantly impaired thymocyte development during the CD4^+^CD8^+^ DP stage^35^. In addition, TCRβ^hi^ CD69^hi^ thymocytes are also diminished in the absence of HDAC3^35^. Thus, similar to NCoR1, HDAC3 may be required for the survival of thymocytes undergoing positive selection. Therefore, HDAC3 may play a role in thymocyte development in both NCoR1-dependent and NCoR1-independent manners.

NCoR1 is known to associate with GR and mediate glucocorticoid-induced repression of GR target gene transcription via the assembly of a GR-NCoR1-HDAC3 complex^36^. It is plausible that GR and NCoR1 may play similar roles in T-cell development. An early study revealed that mice carrying a deletion of GR exon 2 displayed normal thymic and peripheral T-cell development^37^. However, other studies revealed that the deletion of GR exon 2 still yielded a truncated product with the ability to regulate gene expression in a ligand-responsive manner^38, 39^. True GR-deleted mice were later generated by two independent groups by conditionally targeting exon 3^10, 40^. Initially, Baumann et al. claimed that GR-floxed *Lck-Cre* mice had normal thymocyte development^41^, but Mittelstadt et al. later discovered that GR-floxed *Lck-Cre* mice had reduced total thymus cellularity due to a partial block in CD4^−^CD8^−^ DN to DP development^10^. Although the reduction of DP thymocytes was not consistent with our observations in NCoR1-floxed *Lck-Cre* mice, a similar increase in negative selection associated with a decrease in mature CD4 and CD8 thymocytes was also observed in GR-floxed *Lck-Cre* mice. In addition, similar *Bim* upregulation was observed in GR-floxed *Lck-Cre* mice^10^. Thus, NCoR1 is likely to be involved in the regulatory role of GR during thymocyte selection.

Jepsen and colleagues have reported that NCoR1 expression is essential during the progression of thymocyte development from DN3 stage to DP stage^11^. By contrast, the present study revealed that the T-cell-specific deficiency of NCoR1 did not result in DN3 arrest of thymocyte development. Furthermore, the number of total DP thymocytes in NCoR1 cKO mice was similar to that in WT mice. The ineffectiveness of *Ncor1* knockout in the DN thymocytes of *Lck-Cre* mice partially explains this discrepancy. Technically, the previous study is based on the whole-body knockout of NCoR1 and is not able to exclude potential phenotypic contributions of extrinsic effects of NCoR1 deficiency on non-thymocytes. Moreover, because of embryonic lethality, the experiments in that study largely relied on fetal thymic organ cultures rather than live mice. Therefore, the role of NCoR1 in early thymocyte development requires further investigation.

## Methods

### Mice

All the mice were in C57BL/6 J background. The mouse strain carrying flox alleles of NCoR1 was kindly provided by Dr. Johan Auwerx^13^. These mice were bred with transgenic mice harboring *Cre*-*recombinase* driven by the *Lck* or *CD4* promoter to create the following mouse strains: *Ncor1* ^*flox/flox*^ (WT), *Ncor1* ^*flox/flox*^-*Lck*-*Cre* (cKO) or *Ncor1* ^*flox/flox*^-*CD4*-*Cre* (4KO). *Lck*-*Cre* transgenic mice (003802), *CD4*-*Cre* mice (022071), *Bim* ^*−/−*^ mice (004525)*, Rag1* ^*−/−*^ mice (002216), RIP-OVA (Ins2-OVA, 005431) and transgenic mice with the OT-II TCR (003831) were all purchased from the Jackson Laboratory. All mouse strains were bred and maintained under specific pathogen-free (SPF) conditions. Mice, male or female, were used at 6- to 8-week-old, unless otherwise noted. Randomization and blinding strategy was used whenever possible. Mice were killed with carbon dioxide. All mouse experiments followed protocols approved respectively by the Institutional Animal Care and Use Committees in Institut Pasteur of Shanghai and the Salk Institute.

### Flow cytometry

Fluorescence-labeled antibodies for flow cytometry analysis were purchased from eBioscience, Biolegend, and BD Biosciences. Monoclonal antibodies against mouse CD4 (GK15), CD8α (53–6.7), CD69 (H1.2F3), TCRβ (H57-597), MHCI (H-2Kb) (AF6-88.5.5.3), CD5 (53–7.3), CD24 (M1/69), CD62L (MEL-14), Foxp3 (FJK-16s) and TCR Va2 (20.1) were from eBioscience. Antibodies against mouse CD45.1 (A20) and CD45.2 (104) were from BD Biosciences or Biolegend. For intracellular staining of Foxp3, cells were fixed and permeabilized with Fixation/Permeabilization Concentrate and Diluent from eBioscience (00-5223-56). Antibodies all above were used at 1:200 dilution. All the experiments were performed using a BD Fortessa or LSR-II flow cytometer (Becton Dickinson). The data were analyzed using FlowJo (TreeStar).

### Quantitative RT-PCR analysis

Total RNA was extracted from different tissues and cells with TRIzol according to the manufacturer’s instructions (Invitrogen), and cDNA was reverse-transcribed from total RNA using the PrimeScript RT Kit (Takara, RR037). Quantitative PCR was performed using the SYBR^®^ Premix Ex Taq™ II (Takara, RR820) on an ABI 7900HT Fast Real-Time PCR System. The expression of genes was quantitatively normalized to the expression of *Actb* by the change-in-cycling-threshold (ΔΔC_T_) method.

### BM chimeras

Two million T-cell-depleted BM cells from WT (CD45.1^+^CD45.2^+^, or CD45.1^+^) mice and NCoR1-deficient (CD45.2^+^) mice mixed at a ratio of 1:1 were injected into lethally irradiated (600rads, Radsource RS2000 pro) *Rag1* ^−/−^ mice via tail veins. For RIP-OVA chimeras, BM cells from *Ncor1* ^*flox/flox*^-OT-II mice or *Ncor1* ^*flox/flox*^-*Lck*-OT-II mice were injected into lethally irradiated (1000rads, Radsource RS2000 pro) RIP-OVA mice via tail veins, respectively. *Rag1* ^−/−^ and RIP-OVA mice were treated with drinking water containing 2 mg ml^−1^ neomycin sulfate for 1 week before irradiated. Mice were euthanized and analyzed 6 weeks after the BM reconstitution.

### Apoptosis assays

The apoptosis of ex vivo thymocytes or in vitro-activated thymocytes was detected using the FITC Active Caspase-3 Apoptosis Kit (550480, BD Biosciences) or Annexin V (BMS500FI, eBioscience) according to the manufacturers’ guidelines.

### TUNEL staining

Thymi were removed and fixed in 4% paraformaldehyde in phosphate buffer. TUNEL staining was performed using the in situ cell death detection kit according to the manufacturer’s protocol of Boehringer Mannheim. After staining, the sections were further counter-stained with haematoxylin.

### In vitro thymocyte culture

Total or sorted DP thymocytes were cultured with 5 μg ml^−1^ purified anti-CD28 (37.51, eBioscience) in a 96-well plate that was coated with 10 μg ml^−1^ purified anti-TCRβ (H57-597, eBioscience) for the indicated times for apoptosis and quantitative RT-PCR analysis.

### Immunoblot analysis

A total of 10 to 15 million thymocytes were incubated for 30 min on ice with anti-CD3 (5 μg ml^−1^; 145-2C11; eBioscience) plus anti-CD28 (5 μg ml^−1^; 37.51; eBioscience). After crosslinking with goat antibody to hamster immunoglobulin G (20 μg ml^−1^; 55397; MP Biomedicals) at various time points, the cells were lysed and subjected to SDS-PAGE. Antibodies for immunoblot were shown in the Supplementary Table 2.

### Chromatin immunoprecipitation

The chromatin immunoprecipitation (ChIP) assay was carried out essentially following the manufacturer’s protocol (SimpleChIP^®^ Enzymatic Chromatin IP Kit, Agarose Beads, #9002). Each histone modification ChIP was generated using 20 million cells and the following antibodies: 1 μg each of anti-H3ac (06–599, Millipore), anti-H4ac (06–598, Millipore), anti-H3K27ac (ab4729, Abcam), anti-H3K4me1 (ab8895, Abcam) and anti-H3K4me3 (ab8580, Abcam). Rabbit immunoglobulin G (IgG) was used as a control for nonspecific immunoprecipitation of DNA. For quantitative PCR assays, ChIP DNA was amplified for *Bim* using forward (5′-CCACCTCTGCCTCTTAAGTAAC-3′) and reverse (5′-TCCTCCTTTAGGCTCTCCTTAG-3′) primers. The data were analyzed by the following formula: percent (%) input recovery = (100/(input fold dilution/bound fold dilution)) × 2^(input CT−bound CT)^.

### RNA isolation and RNA-Seq

Total mouse thymic RNA was isolated using TRIzol (Invitrogen, 15596026) and the RNeasy Mini Kit with on-column DNase digestion (QIAGEN, 74106). RNA purity was assessed using an Agilent 2100 Bioanalyzer. Sequencing libraries were prepared from 100 ng of total RNA using the TruSeq RNA Sample Preparation Kit v2 (Illumina, RS-122) according to the manufacturer’s protocol.

### Statistical analysis

Statistical analysis of the data (s.d., *t* tests, one- or two-way ANOVA) was performed using GraphPad Prism version 5.0. Sample sizes were designed with adequate power according to the literature and our previous studies. Randomization and blinding strategy was used whenever possible.

### Data availability

The authors declare that the data supporting the findings of this study are available within the article and its supplementary information files, or are available upon reasonable requests to the authors. The RNA-Seq data that support the findings of this study have been deposited at the National Center for Biotechnology Information (NCBI) Sequence Read Archive (SRA) database under the Accession_codes# SRP092624.

Hyperlink:ftp://ftp-trace.ncbi.nlm.nih.gov/sra/review/SRP092624_20170725_133902_b1659515b9d1a59ebbc790e01084a8f0).

## Electronic supplementary material

Supplementary Information

## References

[1] Goldrath AW, Bevan MJ (1999). Selecting and maintaining a diverse T-cell repertoire. Nature.

[2] Palmer E, Naeher D (2009). Affinity threshold for thymic selection through a T-cell receptor-co-receptor zipper. Nat. Rev. Immunol..

[3] Palmer E (2003). Negative selection--clearing out the bad apples from the T-cell repertoire. Nat. Rev. Immunol..

[4] Vrisekoop N, Monteiro JP, Mandl JN, Germain RN (2014). Revisiting thymic positive selection and the mature T cell repertoire for antigen. Immunity.

[5] Jameson SC, Hogquist KA, Bevan MJ (1995). Positive selection of thymocytes. Annu. Rev. Immunol..

[6] Mottis A, Mouchiroud L, Auwerx J (2013). Emerging roles of the corepressors NCoR1 and SMRT in homeostasis. Genes Dev..

[7] Perissi V, Jepsen K, Glass CK, Rosenfeld MG (2010). Deconstructing repression: evolving models of co-repressor action. Nat. Rev. Genet..

[8] Hua G, Paulen L, Chambon P (2016). GR SUMOylation and formation of an SUMO-SMRT/NCoR1-HDAC3 repressing complex is mandatory for GC-induced IR nGRE-mediated transrepression. Proc. Natl Acad. Sci. USA.

[9] Vacchio MS, Ashwell JD (1997). Thymus-derived glucocorticoids regulate antigen-specific positive selection. J. Exp. Med..

[10] Mittelstadt PR, Monteiro JP, Ashwell JD (2012). Thymocyte responsiveness to endogenous glucocorticoids is required for immunological fitness. J. Clin. Invest..

[11] Jepsen K (2000). Combinatorial roles of the nuclear receptor corepressor in transcription and development. Cell.

[12] Mingueneau M (2013). The transcriptional landscape of alphabeta T cell differentiation. Nat. Immunol..

[13] Yamamoto H (2011). NCoR1 is a conserved physiological modulator of muscle mass and oxidative function. Cell.

[14] Li P (2011). Adipocyte NCoR knockout decreases PPARgamma phosphorylation and enhances PPARgamma activity and insulin sensitivity. Cell.

[15] Lesourne R (2009). Themis, a T cell-specific protein important for late thymocyte development. Nat. Immunol..

[16] Wang D (2012). Tespa1 is involved in late thymocyte development through the regulation of TCR-mediated signaling. Nat. Immunol..

[17] Azzam HS (1998). CD5 expression is developmentally regulated by T cell receptor (TCR) signals and TCR avidity. J. Exp. Med..

[18] Yang Y (2004). The E47 transcription factor negatively regulates CD5 expression during thymocyte development. Proc. Natl Acad. Sci. USA.

[19] Gallo EM (2007). Calcineurin sets the bandwidth for discrimination of signals during thymocyte development. Nature.

[20] Fu G (2009). Themis controls thymocyte selection through regulation of T cell antigen receptor-mediated signaling. Nat. Immunol..

[21] Johnson AL (2009). Themis is a member of a new metazoan gene family and is required for the completion of thymocyte positive selection. Nat. Immunol..

[22] Castro JE (1996). Fas modulation of apoptosis during negative selection of thymocytes. Immunity.

[23] Sprent J, Kishimoto H (2002). The thymus and negative selection. Immunol. Rev..

[24] Bouillet P (2002). BH3-only Bcl-2 family member Bim is required for apoptosis of autoreactive thymocytes. Nature.

[25] Sohn SJ, Thompson J, Winoto A (2007). Apoptosis during negative selection of autoreactive thymocytes. Curr. Opin. Immunol..

[26] Ouyang W, Beckett O, Ma Q, Li MO (2010). Transforming growth factor-beta signaling curbs thymic negative selection promoting regulatory T cell development. Immunity.

[27] Cante-Barrett K, Gallo EM, Winslow MM, Crabtree GR (2006). Thymocyte negative selection is mediated by protein kinase C- and Ca2+-dependent transcriptional induction of bim [corrected]. J. Immunol..

[28] Neilson JR, Winslow MM, Hur EM, Crabtree GR (2004). Calcineurin B1 is essential for positive but not negative selection during thymocyte development. Immunity.

[29] Baymaz HI, Karemaker ID, Vermeulen M (2015). Perspective on unraveling the versatility of ‘co-repressor’ complexes. Biochim. Biophys. Acta.

[30] Li P (2013). NCoR repression of LXRs restricts macrophage biosynthesis of insulin-sensitizing omega 3 fatty acids. Cell.

[31] Gardner KE, Allis CD, Strahl BD (2011). Operating on chromatin, a colorful language where context matters. J. Mol. Biol..

[32] Sun Z (2013). Deacetylase-independent function of HDAC3 in transcription and metabolism requires nuclear receptor corepressor. Mol. Cell.

[33] You SH (2013). Nuclear receptor co-repressors are required for the histone-deacetylase activity of HDAC3 in vivo. Nat. Struct. Mol. Biol..

[34] Hsu FC (2015). Histone deacetylase 3 is required for t cell maturation. J. Immunol..

[35] Stengel KR (2015). Histone deacetylase 3 is required for efficient T cell development. Mol. Cell. Biol..

[36] Zamir I (1996). A nuclear hormone receptor corepressor mediates transcriptional silencing by receptors with distinct repression domains. Mol. Cell. Biol..

[37] Purton JF (2002). Glucocorticoid receptor deficient thymic and peripheral T cells develop normally in adult mice. Eur. J. Immunol..

[38] Cole TJ (2001). GRKO mice express an aberrant dexamethasone-binding glucocorticoid receptor, but are profoundly glucocorticoid resistant. Mol. Cell. Endocrinol..

[39] Mittelstadt PR, Ashwell JD (2003). Disruption of glucocorticoid receptor exon 2 yields a ligand-responsive C-terminal fragment that regulates gene expression. Mol. Endocrinol..

[40] Tronche F (1999). Disruption of the glucocorticoid receptor gene in the nervous system results in reduced anxiety. Nat. Genet..

[41] Baumann S (2005). Glucocorticoids inhibit activation-induced cell death (AICD) via direct DNA-dependent repression of the CD95 ligand gene by a glucocorticoid receptor dimer. Blood.

