# Supplementary material for: NCoR1 restrains thymic negative selection by repressing Bim expression to spare thymocytes undergoing positive selection
Source: Nat Commun. 2017 Oct 16;8:959. doi: 10.1038/s41467-017-00931-8 (PMC5643384; doi:10.1038/s41467-017-00931-8)
Supplement: Supplementary file 1 — Supplementary Information [file 41467_2017_931_MOESM1_ESM.pdf]

Supplementary Figure 1

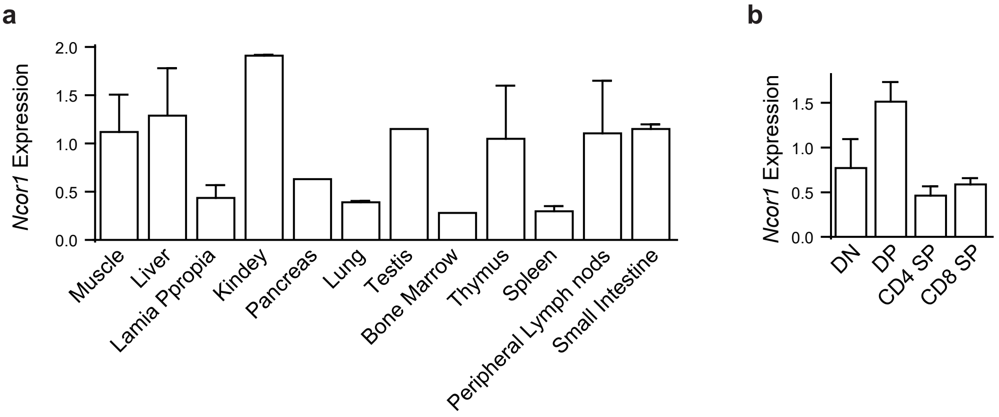

Supplementary Figure 1. *Ncor1* expression profiles in different tissues and thymocyte subsets. (**a**) Quantitative RT-PCR analysis of *Ncor1* mRNA expression in various tissues(n=3) and (**b**) in the indicated thymocyte subsets (n=3, repeat 2 times) sorted using flow cytometry. DN, $CD4^-CD8^-$ double-negative thymocytes; DP, $CD4^+CD8^+$ double-positive thymocytes; CD4 SP, $CD4^+$ single-positive thymocytes; CD8 SP, $CD8^+$ single-positive thymocytes.

Supplementary Figure 2

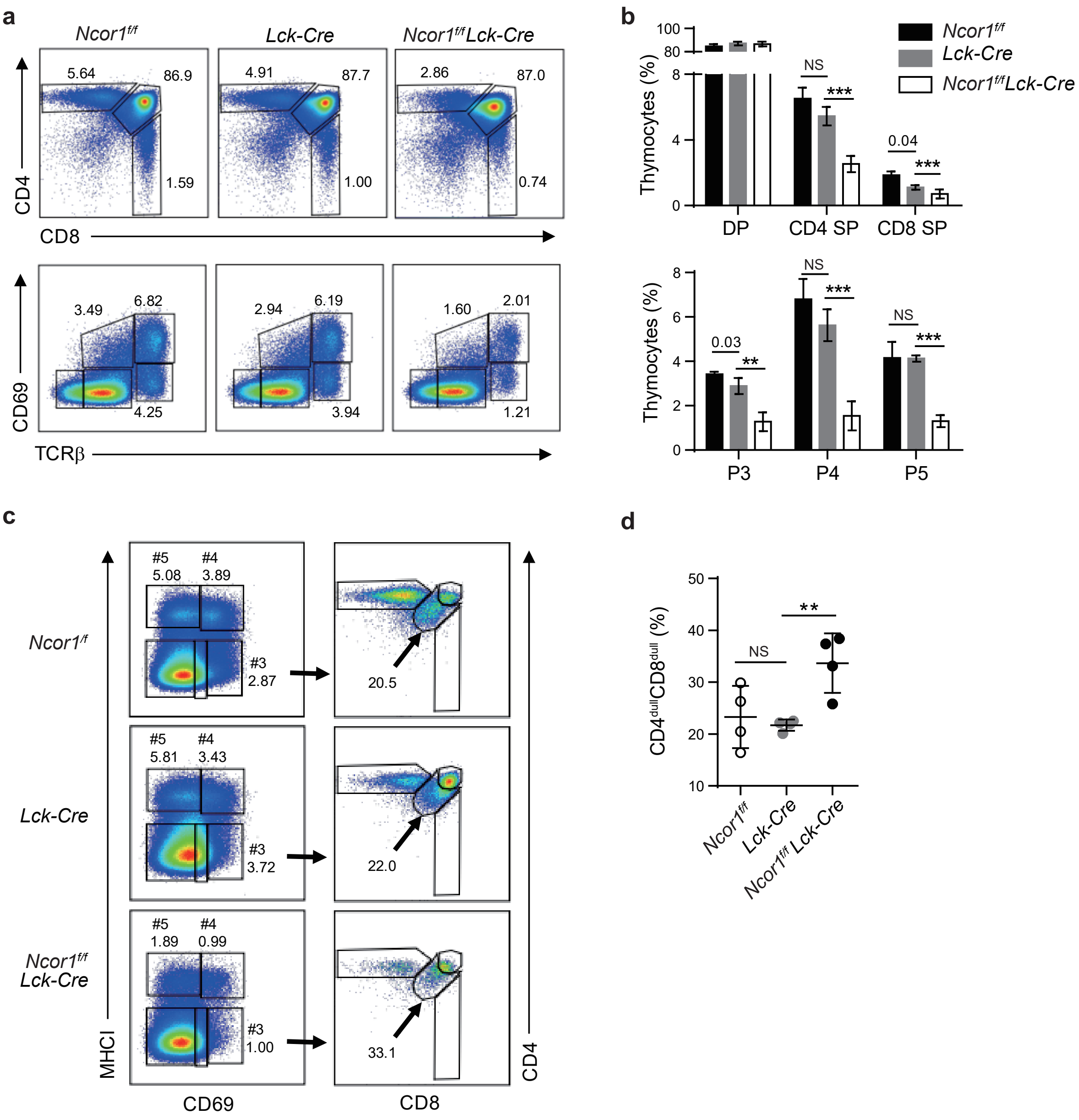

Supplementary Figure 2. Thymocyte profiles in *Ncor1*$^{flox/flox}$, *Lck-Cre* and *Ncor1*$^{flox/flox}$*Lck-Cre* mice. (**a**) Surface expression of CD4 versus CD8 (up) and CD69 versus TCRβ (down) on total thymocytes (n=4 for each group) detected by flow cytometry. (**b**) Quantification of different thymocyte subsets in (**a**). (**c**) Flow cytometry analysis of 5 sub-populations (left) by MHCI and CD69 expression on total thymocytes (n=4 for each group, respectively). The right panel shows CD4 and CD8 expression on gated population #3. (**d**) The proportion of CD4^{dull}CD8^{dull} subsets in population #3. Statistical significance was analysed using the two-tailed Student's t test (NS, not significant; ** P<0.01; *** P<0.001).

Supplementary Figure 3

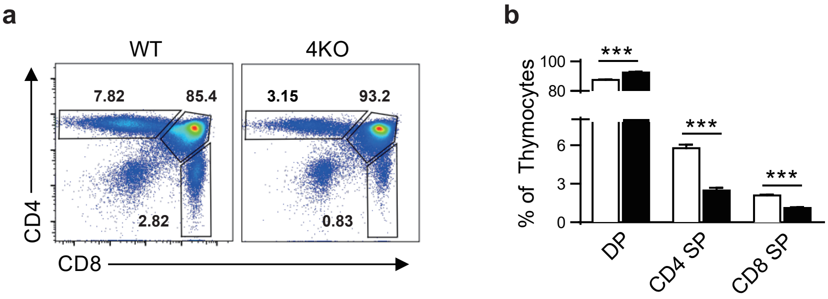

Supplementary Figure 3. Thymocyte profiles in *Ncor1^flox/flox^CD4-Cre* mice. (**a**) Surface expression of CD4 and CD8 on thymocytes from *Ncor1*$^{flox/flox}$*CD4-Cre* (4KO) mice and *Ncor1*$^{flox/flox}$ (WT) mice (n=3 to 5). (**b**) Quantification of different thymocyte subsets in (**a**). DP, $CD4^+CD8^+$ double-positive thymocytes; CD4 SP, $CD4^+$ single-positive thymocytes; CD8 SP, $CD8^+$ single-positive thymocytes. Data are representative of three independent experiments. Statistical significance was analysed using the two-tailed Student's t test (*** P<0.001).

Supplementary Figure 4

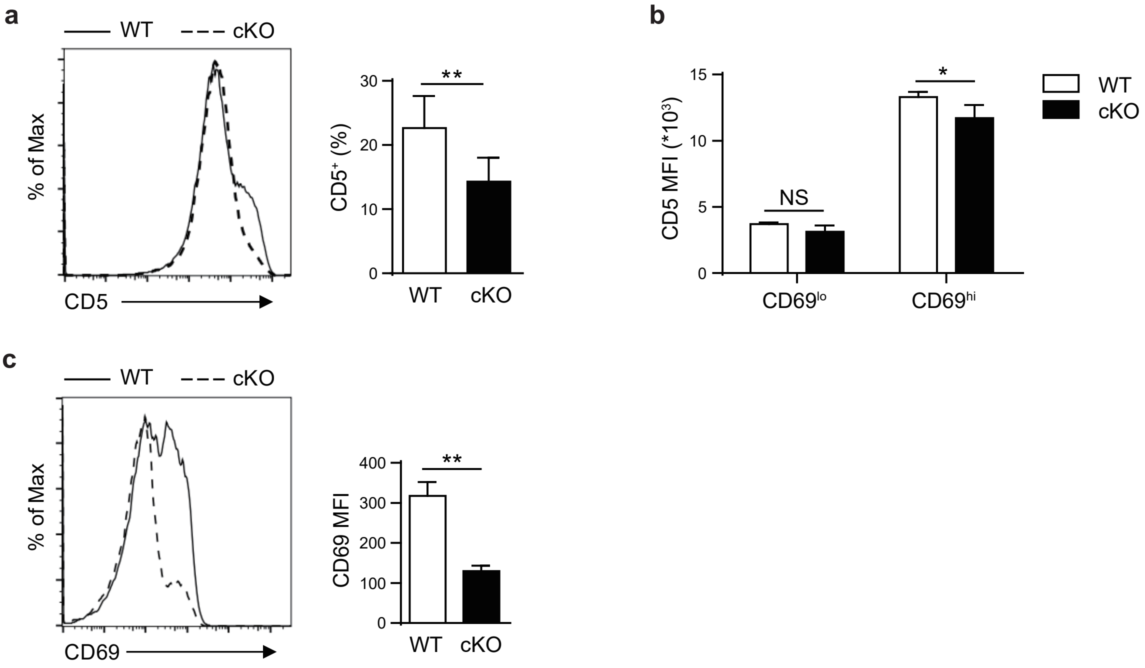

Supplementary Figure 4. CD5 and CD69 expression in NCoR1-deficient thymocytes. (**a**) Flow cytometry analysis of CD5 expression (left) on total thymocytes from NCoR1-deficient (cKO) and wild type (WT) mice (n=3 to 5) and quantification of the CD5-positive sub-population (right). (**b**) Mean fluorescence intensity of CD5 on CD69^{lo} and CD69^{hi} DP（CD4^{+}CD8^{+} double-positive) thymocytes from cKO (n=4) and WT (n=4) mice. (**c**) The histogram plot (left) and the mean fluorescence intensity (right) of CD69 expression on total thymocytes from cKO and WT OT-II TCR-transgenic mice (n=3) detected by flow cytometry. Data are representative of two independent experiments. Statistical significance was analysed using the two-tailed Student's t test (NS, not significant, ** P<0.01).

Supplementary Figure 5

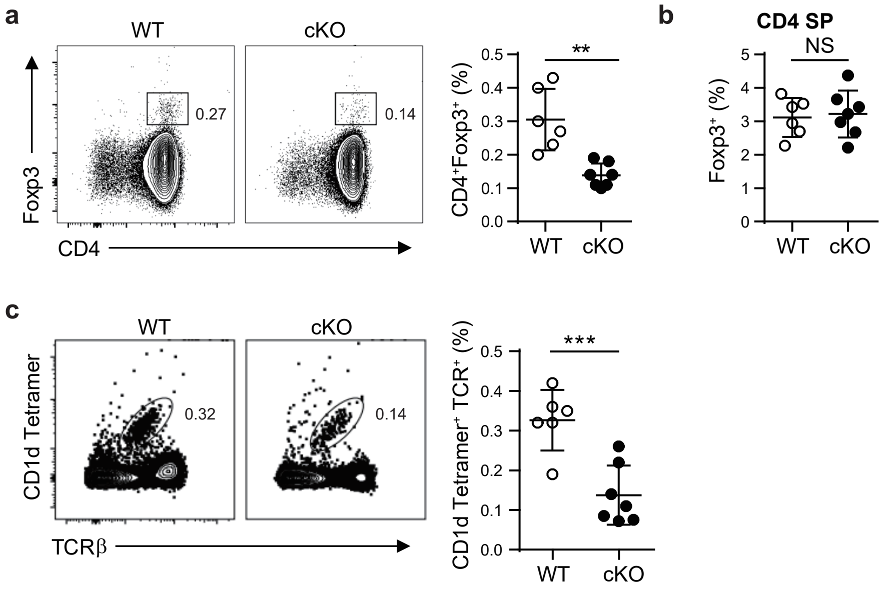

Supplementary Figure 5. Development of both Treg cells and iNKT cells is defective in the thymus of NCoR1-deficient mice. (**a**) Foxp3 expression in total thymocytes from NCoR1-deficient (cKO) (n=7) and wild type (WT) (n=6) mice by intracellular staining (left) and quantification of Foxp3^{+} cells (right). (**b**) Quantification of Foxp3^{+} cells in CD4^{+} single-positive (CD4 SP) WT and cKO thymocytes in (**a**). (**c**) CD1d Tetramer and TCRβ expression in total thymocytes from WT (n=6) or cKO (n=7) mice (left) by flow cytometry and quantification of CD1d Tetramer^{+}TCRβ^{+} cells (right). Statistical significance was analysed using the two-tailed Student's t test (NS, not significant; ** P<0.01; *** P<0.001).
.

Supplementary Figure 6

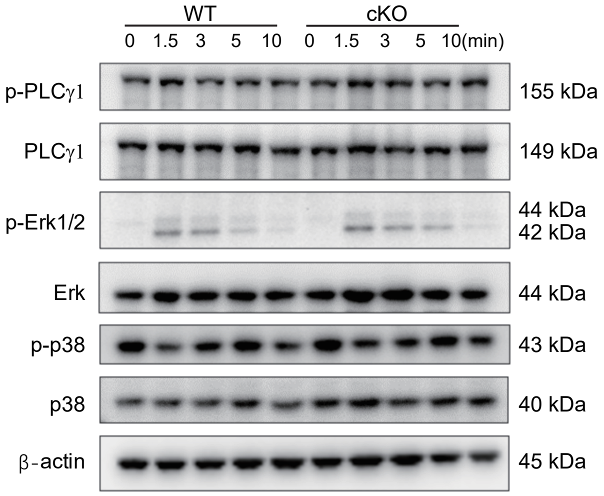

Supplementary Figure 6. TCR signaling in NCoR1-deficient thymocytes. Immunoblot analysis of phosphorylated (p)-PLC-γ1, p-Erk1/2, p-p38 and total protein of PLC-γ1, Erk and p38 in lysates from wild type (WT) and NCoR1-deficient (cKO) thymocytes without or with stimulation *in vitro* for the indicated amounts of time. β-actin served as a loading control. Data are representative of there independent experiments.

Supplementary Figure 7

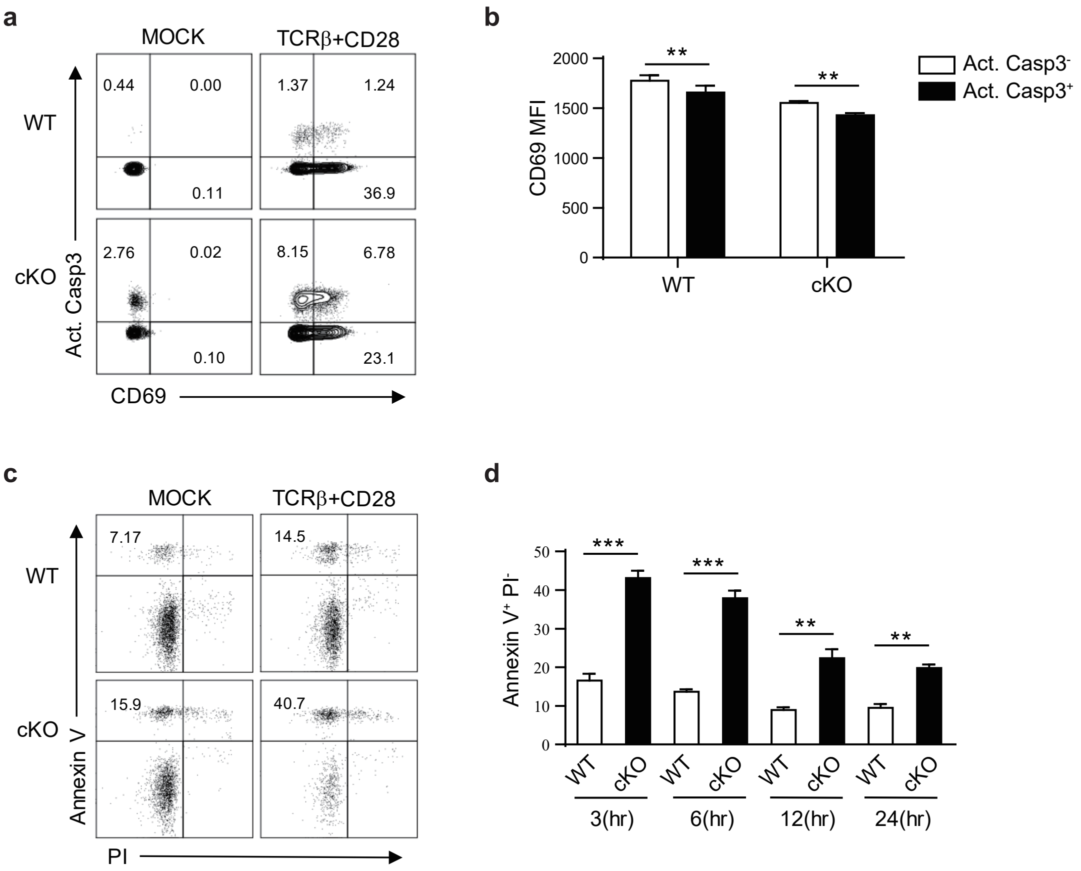

Supplementary Figure 7. NCoR1-deficient thymocytes are prone to apoptosis. (**a**) The surface marker CD69 and intracellular staining of the active caspase-3 (Act.Casp.3) detected by flow cytometry in total NCoR1-deficient (cKO) (n=3) and wild type (WT) (n=3) thymocytes stimulated or not with TCR β and CD28 for 6 hr. (**b**) The mean fluorescence intensity of CD69 expression in Act.Casp3^{+} and Act.Casp3^{-} thymocytes in (**a**). (**c**) Flow cytometric staining of Annexin V and propidium iodide (PI) on WT and cKO thymocytes stimulated *in vitro* for 6 hr (n=3 to 5). (**d**) Quantification of the apoptotic thymocytes shown in (**c**). Data are representative of two independent experiments. Statistical significance was analysed using the two-tailed Student's t test (** P<0.01; *** P<0.001).

Supplementary Figure 8

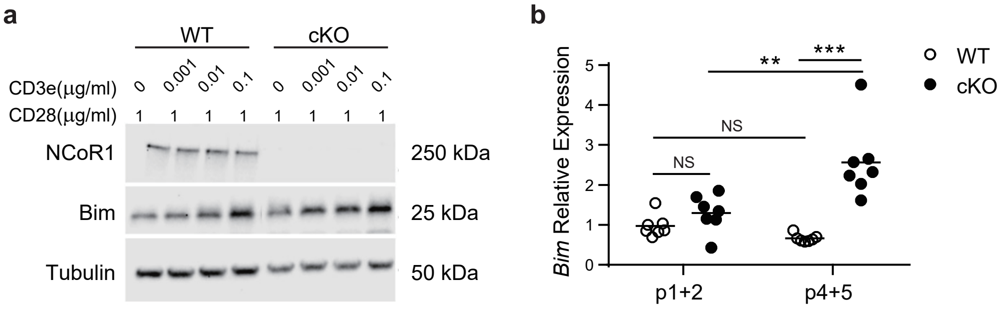

Supplementary Figure 8. Bim expression in TCR signalling stimulated thymocytes *in vitro* and in sorted non-activated and activated thymocytes *in vivo.* (**a**) Bim protein levels in NCoR1-deficient (cKO) and wild type (WT) thymocytes stimulated with different concentrations of anti-TCRβ. (**b**) Non-activated thymocytes (population 1 + 2, p1+2) and activated thymocytes (population 4 + 5, p4+5) were sorted by the surface expression of CD69 and TCRβ on total WT (n=7) and cKO (n=7) thymocytes according to the gating in Fig. 2a. *Bim* mRNA levels in these sorted thymocytes were analysed by quantitative RT-PCR. Statistical significance was analysed using the two-tailed Student's t test (NS, not significant, ** P<0.01; *** P<0.001).

Supplementary Figure 9

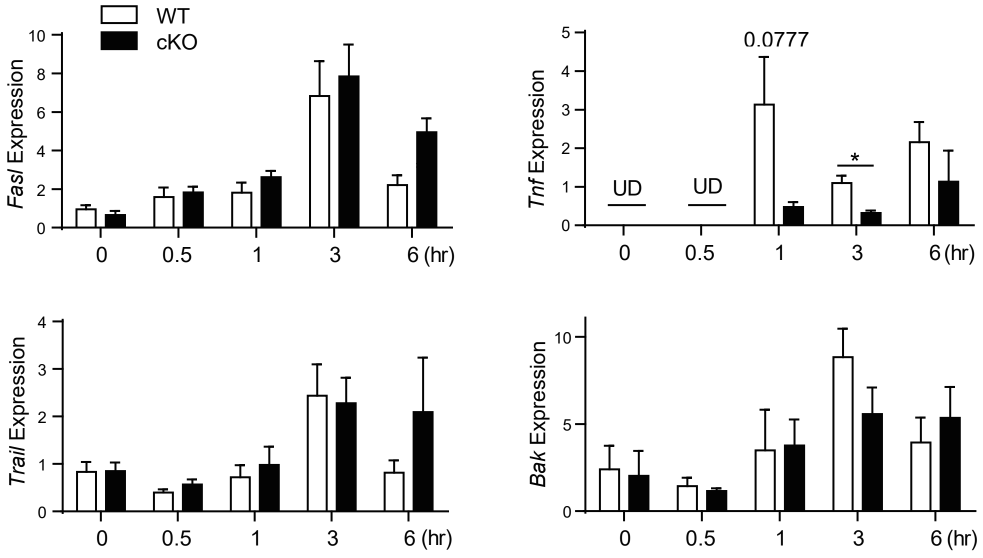

Supplementary Figure 9. Expression of proapoptotic genes in enriched DP thymocytes. Quantitative RT-PCR analysis of *Fasl*, *Tnf*, *Trail*, and *Bak* mRNA levels in enriched NCoR1-deficient (cKO) and wild type (WT) DP ($CD4^{+}CD8^{+}$ double-positive) thymocytes activated *in vitro* for the indicated time points. Data are representative of two independent experiments with four mice per genotype. The number indicates the p value. Statistical significance was analysed using the two-tailed Student's t test

Supplementary Figure 10

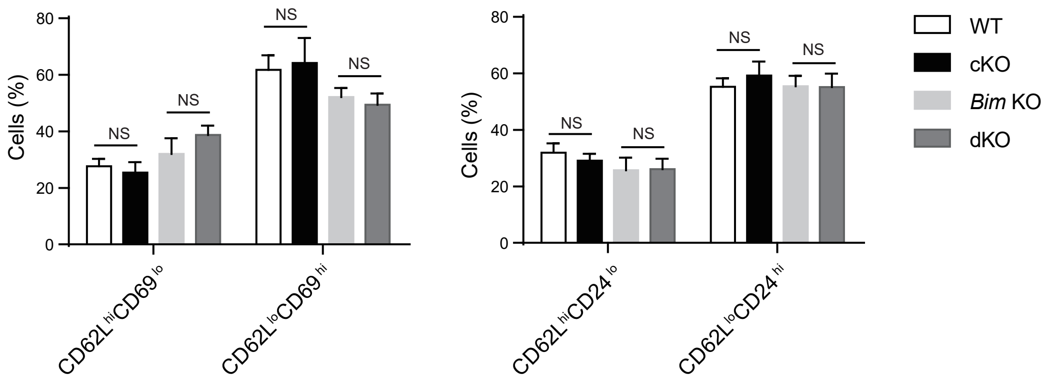

Supplementary Figure 10. Proportions of mature (CD62L^{hi}CD69^{lo} or CD62L^{hi}CD24^{lo}) and immature (CD62L^{lo}CD69^{hi} or CD62L^{lo}CD24^{hi}) subsets in WT (n=4) , cKO (n=4) , *Bim* KO (n=4) and dKO (n=5) thymocytes. Flow cytometry analysis and gating of mature or immature subsets were performed as shown in Fig. 6c. WT, wild type; cKO, NCoR1-deficient; dKO, *Ncor1* and *Bim* double knockout.Statistical significance was analysed using the two-tailed Student's t test (NS, not significant).

Supplementary Figure 11

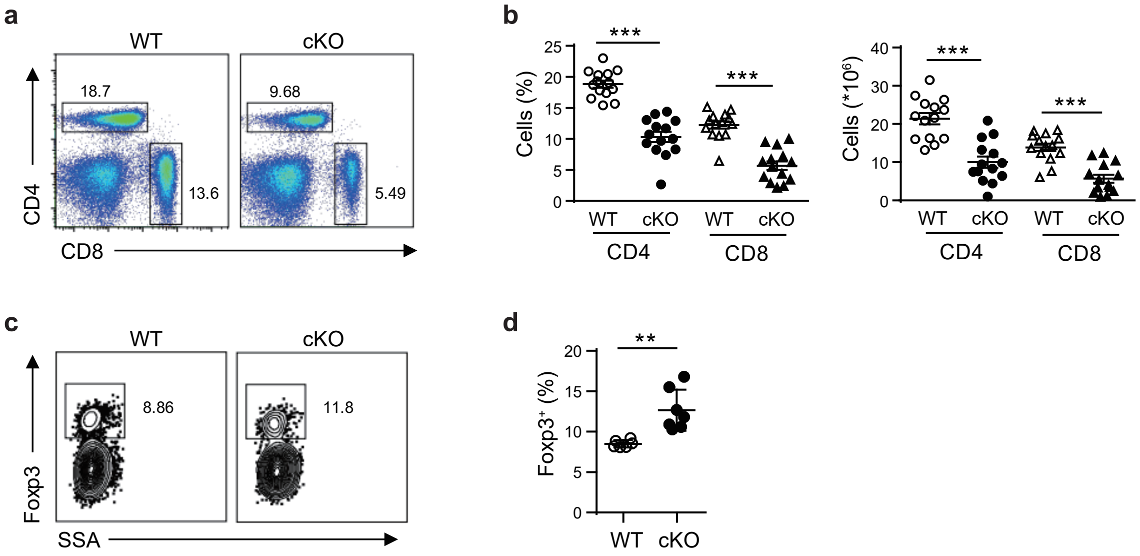

Supplementary Figure 11. Loss of NCoR1 reduces peripheral CD4 and CD8 T cells in the spleen but increases the proportion of Tregs. (**a**) The surface expression of CD4 and CD8 on NCoR1-deficient (cKO) (n=4) and wild type (WT) (n=4) splenocytes detected by flow cytometry. (**b**) The percentages (left) or numbers (right) of CD4 and CD8 T cells gated in (**a**). Data were generated from three independent experiments. (**c**) Intracellular expression of Foxp3 in $CD4^+$ $TCR\beta^+$ cKO (n=6) and WT (n=6) splenocytes detected by flow cytometry. (**d**) Quantification of the $Foxp3^+CD4^+$ T cells in (**c**). Data are representative of three independent experiments with more than three mice per group. Statistical significance was analysed using the two-tailed Student's t test (** P<0.01; *** P<0.001).

Supplementary Figure 12

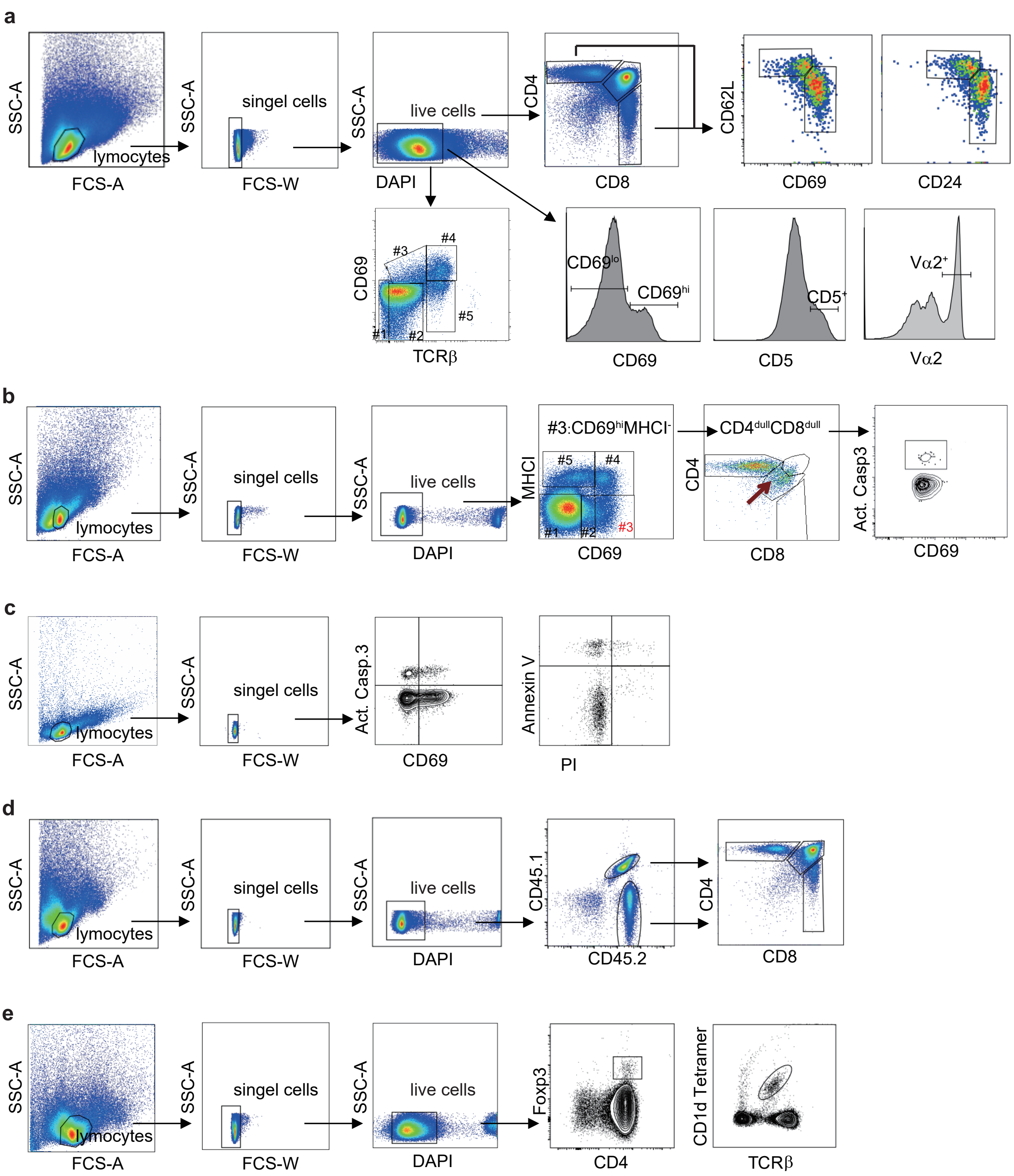

Supplementary Figure 12. Gating strategies for flow cytometry analyses. Representive FACS plots showing: (**a**) Gating strategy for CD4, CD8, CD69, TCRβ, CD5, and Vα2 (for OTII background) expression. (**b**) Gating strategy for intracellular Act.Casp.3 in CD69^{hi}MHCI^{-}CD4^{dull}CD8^{dull} thymocytes. (**c**) Gating strategy to detect apoptosis of thymocytes stimulated *in vitro*.(**d**) Gating strategy to analyze thymocytes profiling in bone marrow chimeras. (**e**) Gating strategy to analyze T regulatory cells and invariant natural killer T cells in thymus.

Supplementary Figure 13

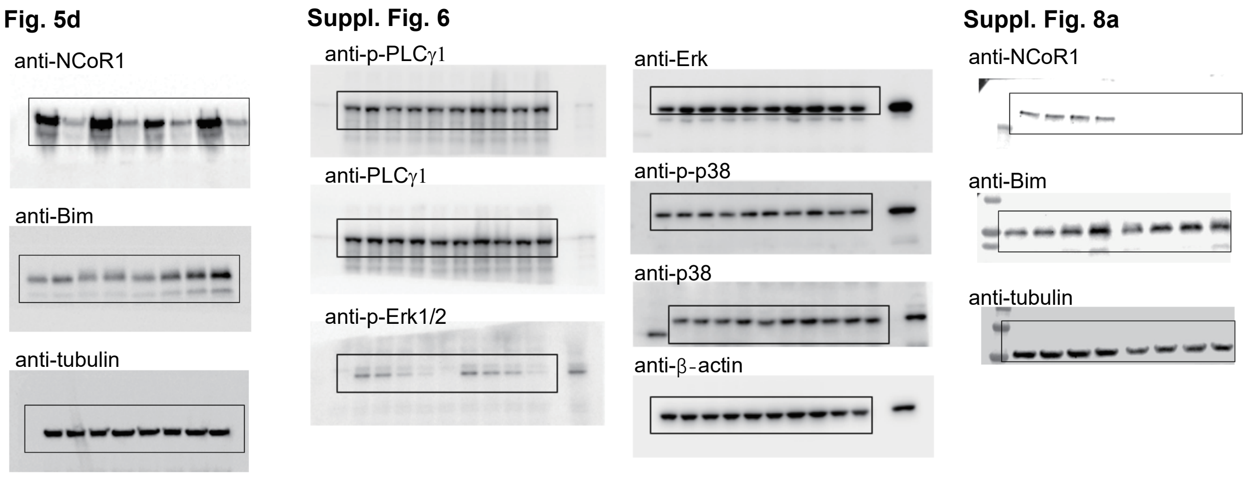

Supplementary Figure 13. The uncropped scans of western blots from Figure 5, Supplemenary Figure 6 and 8a.

Supplemental Table 1. Genes expressed differentially in thymocytes by RNA-seq

In steady state thymocytes

Gene	cKO/WT (ex vivo)
	log2(fold_change)
Gm7125	infinite
Gm6594	infinite
AC163653.5	infinite
Gh	infinite
AC158672.3	infinite
Sfn	1.41297
Spsb1	1.2823
St3gal6	1.25667
Wnt8b	1.24523
Gm8186	1.10604
Dusp4	1.08605
Ifngr1	1.05791
Tbl1xr1	0.954922
Cdk5r1	0.926474
Bcl6	0.918982
Mbnl1	0.853401
Dusp5	0.831608
Slc9a9	0.798935
Scd2	0.785073
Cd74	0.72575
Pik3r1	0.725251
Pik3cg	0.720207
Gimap6	0.692412
Ctsl	0.683771
Lig4	0.680034
Tespa1	0.670143
Tubb2b	0.627266
Abcg1	0.595489
Esyt1	-0.632325
Ltb	-0.645846
Gpr56	-0.670893
Rpl23a	-0.674669
Nrp1	-0.728366
Ntn1	-0.742279
Smoc1	-0.791651

Gene	cKO/WT (ex vivo)
	log2(fold_change)
Adcy7	-0.801285
Itgb7	-0.82898
Cxxc5	-0.833748
Als2cl	-0.847795
Mllt6	-0.866141
Cnn2	-0.918308
Pdcd1	-0.949894
Cd53	-0.964088
St3gal1	-1.00801
Slc6a19	-1.0084
Shisa5	-1.01297
Sema7a	-1.0856
AB124611	-1.08824
H2-K1	-1.10853
Fgf13	-1.16045
Padi4	-1.1741
AC165246.1	-1.21028
Ccr7	-1.26486
Slfn1	-1.30495
H2-Q7	-1.31484
Ephx1	-1.32313
Ctsw	-1.32528
Nkg7	-1.32796
Xrra1	-1.36339
Jup	-1.40723
Phlda1	-1.42058
Paqr7	-1.57394
Ccnd2	-1.59108
Lfng	-1.67108
Cd7	-1.67425
Gm10499	-1.73999
Sdc4	-1.93547
n-R5-8s1	-2.25768
Cd200	-3.59722
Gm11223	-4.56491

In activated thymocytes

Gene	KO/WT (3h activation)
	log2(fold_change)
Gm7125	infinite
Gm6594	infinite
Ighv1-49	infinite
AC160982.1	2.03594
Sfn	1.3304
Acoxl	1.14459
Tbl1xr1	1.06335
Scd2	1.06322
Il12rb1	0.956431
Dusp4	0.913305
ERDR1	0.896455
Gimap9	0.89101
Bcl6	0.852289
Abcg1	0.841595
Ptger4	0.739682
Ldlr	0.732622
Gimap8	0.719569
Gramd3	0.707089
Bcl2l11	0.673482
Mr1	0.651132
Mbnl1	0.638582
Ccnd3	0.630751
Ctsl	0.612296
Ccr4	0.592412
Irf4	-0.655463
Cd53	-0.671309
Ntn1	-0.739517
Icos	-0.75102
Gpr56	-0.800305

Gene	KO/WT (3h activation)
	log2(fold_change)
B4galnt1	-0.800943
Myc	-0.803976
Gm14085	-0.814945
Slc6a19	-0.820457
Ccr7	-0.823115
H2-K1	-0.839568
Fam134b	-0.856552
Adora2a	-0.886581
Tnf	-0.955403
Cxxc5	-0.955899
Evl	-1.04317
Pdcd1	-1.07871
H2-Q7	-1.11737
Cd83	-1.25182
Ifi27l2a	-1.3544
Cited4	-1.38267
Lad1	-1.43564
Tagap	-1.43739
Egr3	-1.63327
Xcl1	-1.71541
Lfng	-1.89111
Tnfrsf9	-2.02836
Rgs16	-2.09375
Ccnd2	-2.23925
n-R5-8s1	-2.32324
Sh3gl3	-2.4747
Apbb2	-3.47654
Gm11223	-3.80225

Supplemental Table 2. Antibodies Information

Antibodies for Flow cytometry analysis

Antibody	Clone	Dilution	Company
CD4	GK15	1:200	eBioscience
CD8α	53-6.7	1:200	eBioscience
CD69	H1.2F3	1:200	eBioscience
TCRβ	H57-597	1:200	eBioscience
MHCI	AF6-88.5.5.3	1:200	eBioscience
CD5	53-7.3	1:200	eBioscience
CD24	M1/69	1:200	eBioscience
CD62L	MEL-14	1:200	eBioscience
CD45.1	A20	1:200	BD Bioscience
CD45.2	104	1:200	BD Bioscience
Foxp3	FJK-16s	1:200	eBioscience
Vα2	20.1	1:200	eBioscience

Antibodies for Immunoblot analysis

Antibody	Code	Dilution	Company
anti-rabbit-NCoR1	5948S	1:1000	Cell Signaling Technology
anti-rabbit-Bim	2933S	1:1000	Cell Signaling Technology
anti-rabbit-p-PLCγ1	2821S	1:1000	Cell Signaling Technology
anti-mouse-PLCγ1	ab76155	1:1000	Abcam
anti-mouse-p-Erk1/2	sc-7383	1:500	Santa Cruz Biotechnology
anti-rabbit-Erk	sc-93	1:2000	Santa Cruz Biotechnology
anti-rabbit-p-p38	9211S	1:1000	Cell Signaling Technology
anti-rabbit-p38	sc-728	1:2000	Santa Cruz Biotechnology
anti-rabbit-β-actin	4970S	1:2000	Cell Signaling Technology
anti-rabbit-tublin	CP06	1:2000	EMD Millipore
Goat anti-rabbit-IgG(H+L)	111-035-003	1:3000	Jackson Immuno&Research
Donkey anti-mouse-IgG(H+L)	715-035-150	1:3000	Jackson Immuno&Research

Antibodies for CHIP analysis

Antibody	Code	Dilution	Company
anti-NCoR1	5948S	1 g per IP	Cell Signaling Technology
anti-H3ac	06-599	1 g per IP	Millipore
anti-H4ac	06-598	1 g per IP	Millipore
anti-H3K27ac	ab4729	1 g per IP	Abcam
anti-H3K4me1	ab8580	1 g per IP	Abcam
anti-H3K4me3	ab6002	1 g per IP	Abcam
IgG	3900S	1 g per IP	Cell Signaling Technology

Supplemental Table 3. Primer for Quantitative RT-PCR analysis

Ncor1	Forward	5'-TGCGTCAGCTTTCTGTGA TTCCACC-3'
	Reverse	5'-TGA TTTCTGCCTCTGCGTTTTCCA-3'
Bim	Forward	5'-CGACAGTCTCAGGAGGAACC-3'
	Reverse	5'-CCTTCTCCATACCAGACGGA-3'
Fasl	Forward	5'-TGAATTACCCATGTCCCCAG-3'
	Reverse	5'-AAACTGACCCTGGAGGAGCC-3'
Tnf	Forward	5'-CATCTTCTCAAAATTCGAGTGACAA-3'
	Reverse	5'-TGGGAGTAGACAAGGTACAACCC-3'
Trail	Forward	5'-ATGATGGTGATTTGCATAGTGCT-3'
	Reverse	5'-AGCTGCTTCATCTCGTTGGTG-3'
Bak	Forward	5'-AATGGCATCCGGACAAGGAC-3'
	Reverse	5'-TGTTCCTGCTGGTGGAGGTA-3'
Actb	Forward	5'-GGCTGTATTCCCCTCCATCG-3'
	Reverse	5'-CCAGTTGGTAACAATGCCATGT-3'
